# Editorial: Future horizons in diabetes: integrating gut microbiota, AI, and personalized care

**DOI:** 10.3389/fendo.2026.1875810

**Published:** 2026-05-18

**Authors:** Nazarii Kobyliak, Tetyana Falalyeyeva

**Affiliations:** 1 Bogomolets National Medical University, Kyiv, Ukraine; 2 Medical Laboratory CSD, Kyiv, Ukraine; 3 Taras Shevchenko National University of Kyiv, Kyiv, Ukraine

**Keywords:** artificial intelligence, diabetes, gut microbiota, personalized care, type 2 diabetes mellitus

Diabetes is still a major health problem worldwide. Even with new medicines, better ways to track the disease, and efforts to prevent it, an increasing number of people are experiencing type 2 diabetes (T2D) and related health issues. This situation is becoming more complicated by the consequences of the COVID-19 pandemic, a global increase in sedentary lifestyles, and the widespread adoption of the “Western diet” (1–3). Whereas diabetes is complex and has many causes, doctors need to treat it more comprehensively and in a more personalized way rather than just focusing on blood sugar levels. Therefore, treatment should consider all aspects of a patient’s health and lifestyle to provide the best possible care. Recent research has indicated a promising future for diabetes care, with new ideas and discoveries that could change how we manage this condition, particularly through microbiota modulation (4–6).

The Research Topic “Future Horizons in Diabetes: Integrating the Gut Microbiota, AI, and Personalized Care” was designed to demonstrate new concepts and translational advances that could reshape the future of diabetes management. The 17 articles on this topic examine the latest advances in diabetes research, covering everything from tiny microbes to digital technologies and innovative treatments. What is clear is that diabetes is more than just a problem with how our bodies process sugar – it is a complex condition that affects many different parts of our bodies and needs a comprehensive approach to treat it effectively. By combining experts from many different fields, we can develop new and better ways to prevent, diagnose, and manage diabetes, especially T2D, and improve the lives of people living with this condition.

Gender differences in diabetes development have been reported. Compared with males, females with prediabetes, despite having worse dyslipidemia and more visceral fat, often have better glucose tolerance and insulin sensitivity. These differences may stem from variations in fat distribution and inflammation. Males are more prone to early prediabetic issues such as insulin resistance and fatty liver, regardless of obesity (Hüttl et al., Zhang et al.). Diabetes can lead to problems with the nerves, blood vessels, and digestive system (Xu et al., Shaienko). For instance, individuals with diabetes are at an increased risk of developing liver disease, experiencing muscle weakness, and suffering from nerve damage (Zhang et al., Wang et al.). They may also face issues with blood circulation in their feet and legs, as well as with digestion (Shaienko). Researchers are investigating these various complications to better understand how they are interconnected with diabetes. Their goal is to find new treatment approaches that consider all the different systems in the body that are affected by the disease. This perspective emphasizes viewing diabetes as a whole-body health issue rather than focusing solely on the pancreas or blood sugar levels. By understanding how diabetes affects various bodily systems, doctors and researchers can develop more effective treatments that address all related issues. This could lead to improved health outcomes for people living with diabetes and reduce the risk of long-term complications.

The gut microbiota is becoming a key player in understanding metabolic health and disease. Research has shown that the balance of gut microbes is related to genes, diet, inflammation, and insulin resistance. For example, articles addressing dietary fiber interventions in patients with gestational diabetes, microbial signatures in patients with type 3c diabetes, and the relationship between gut dysbiosis and diabetic neuropathy have been published (Soto et al., Zhang et al., Horiachok et al.). There is also growing interest in how genes influence the body’s interactions with gut microbes (Liang et al.). The effects described suggest that modulating the gut microbiota could be an important strategy for treating T2D. This could involve changing the diet; taking prebiotics, probiotics, or postbiotics; or using other approaches that affect gut microbes, such as increasing physical activity (Savytska et al., Wang et al., Zhang et al.). By doing so, doctors may be able to create personalized treatment plans that account for an individual’s unique gut ecosystem.

This Research Topic examines how artificial intelligence (AI) and data-driven medicine can work together to enhance healthcare systems. With the vast amount of complex data generated by these systems, AI can assist in making predictions, diagnosing diseases, and developing personalized treatment plans. Research in this field has demonstrated how smart decision-making tools, machine learning, and innovative diagnostic methods can be utilized to manage blood sugar levels and improve treatment accuracy (7) (Wang et al., Amuti et al.. Zhang et al.). By integrating biological markers with computer analysis, these technologies can help identify individuals at risk, increase treatment effectiveness, and reduce the workload for both doctors and patients (7).

New treatments for diabetes are being explored, offering much hope. Scientists are investigating innovative ways to lower blood sugar levels and are making significant progress (Trzhetsynskyi et al., Yang et al.). How cells communicate with one another and how genes can affect diabetes are being studied (Qiao et al.). This strategy has the potential to lead to new treatments that can greatly benefit people living with diabetes. It is exciting to see how science can be harnessed to improve lives. By combining basic research with clinical trials, researchers can transform their discoveries into effective treatments that enhance health. This represents a major advancement in the care of individuals with diabetes.

Importantly, the breadth of topics represented in this Research Topic illustrates the critical role of interdisciplinary collaboration in modern diabetology. Continued progress increasingly depends on the integration of endocrinology, gastroenterology, nutrition science, molecular medicine, bioinformatics, and clinical practice. By combining expertise across disciplines, researchers and clinicians can develop more comprehensive and effective strategies for one of the most challenging chronic diseases of our time.

As topic editors, we extend our sincere appreciation to all the authors for their high-quality submissions, to the reviewers for their thoughtful and constructive assessments, and to the editorial staff for their continuous support during the publication process. Their combined efforts made this Research Topic possible. We hope that the studies presented here will stimulate further investigation, strengthen scientific partnerships, and encourage continued innovation in precision diabetology. The future of diabetes care lies in uniting biological insight, digital technologies, and personalized medicine, and this Research Topic represents an important step in that direction.
